# Resolving single-cell heterogeneity from hundreds of thousands of cells through sequential hybrid clustering and NMF

**DOI:** 10.1093/bioinformatics/btaa201

**Published:** 2020-03-24

**Authors:** Meenakshi Venkatasubramanian, Kashish Chetal, Daniel J Schnell, Gowtham Atluri, Nathan Salomonis

**Affiliations:** b1 Department of Electrical Engineering and Computer Science, University of Cincinnati; b2 Division of Biomedical Informatics, Cincinnati Children’s Hospital Medical Center; b3 Department of Biomedical Informatics, University of Cincinnati, Cincinnati, OH 45267, USA

## Abstract

**Motivation:**

The rapid proliferation of single-cell RNA-sequencing (scRNA-Seq) technologies has spurred the development of diverse computational approaches to detect transcriptionally coherent populations. While the complexity of the algorithms for detecting heterogeneity has increased, most require significant user-tuning, are heavily reliant on dimension reduction techniques and are not scalable to ultra-large datasets. We previously described a multi-step algorithm, Iterative Clustering and Guide-gene Selection (ICGS), which applies intra-gene correlation and hybrid clustering to uniquely resolve novel transcriptionally coherent cell populations from an intuitive graphical user interface.

**Results:**

We describe a new iteration of ICGS that outperforms state-of-the-art scRNA-Seq detection workflows when applied to well-established benchmarks. This approach combines multiple complementary subtype detection methods (HOPACH, sparse non-negative matrix factorization, cluster ‘fitness’, support vector machine) to resolve rare and common cell-states, while minimizing differences due to donor or batch effects. Using data from multiple cell atlases, we show that the PageRank algorithm effectively downsamples ultra-large scRNA-Seq datasets, without losing extremely rare or transcriptionally similar yet distinct cell types and while recovering novel transcriptionally distinct cell populations. We believe this new approach holds tremendous promise in reproducibly resolving hidden cell populations in complex datasets.

**Availability and implementation:**

ICGS2 is implemented in Python. The source code and documentation are available at http://altanalyze.org.

**Supplementary information:**

Supplementary data are available at *Bioinformatics* online.

## 1 Introduction

Recent advances in single-cell RNA-sequencing (scRNA-Seq) provide exciting new opportunities to understand cellular and molecular diversity in healthy tissues and disease. With the rapid growth in scRNA-Seq, numerous computational applications have been developed that address diverse technical challenges such as measurement noise/accuracy, data sparsity and high dimensionality to identify cell heterogeneity within potentially complex cell populations. Most software applications consist of a shared set of steps, including: (i) gene filtering, (ii) expression normalization, (iii) dimension reduction and (iv) clustering (Andrews and Hemberg, 2018). While the specific algorithms and options used for these steps varies significantly among applications, most approaches rely heavily on dimension reduction techniques, such as PCA, t-SNE and UMAP to select features and define cell populations. As noted by others (Andrews and Hemberg, 2018), the reliance on such techniques has several limitations, including insensitivity to non-linear sources of variance (e.g. when defined using PCA), loss of global structure due to a focus on local information (t-SNE) (Maaten and Hinton, 2008) and inability to scale to high-dimensions (UMAP) (McInnes and Healy, 2018), resulting in a significant loss of information during projection.

While a number of methods exists to identify clusters from large lower dimensional projections, including DBSCAN, K-means, affinity propagation, Louvain clustering and spectral clustering, these as well as other approaches require proper hyperparameter tuning. Identifying these parameters is non-intuitive and often requires multiple rounds of analysis. To address this concern, consensus-based approaches that consider the results from multiple runs with different parameters have been developed, such as SC3 (Kiselev *et al.*, 2017); however, the ultimate selection of the parameters remains user dependent and is not automated.

A separate but related challenge is the analysis of cell atlases, with potentially hundreds of thousands of cells and samples collected from different donors. Multiple joint-alignment methods have been developed to address such challenges, including Seurat3, conos, Scanorama, Biscuit, scVI, LIGER, scMerge and Harmony (Azizi *et al.*, 2018; Hie *et al.*, 2019; Korsunsky *et al.*, 2019; Lin *et al.*, 2019; Lopez *et al.*, 2018; Welch *et al.*, 2019). While such tools can reduce the contribution of technical artifacts, further minimizing the impact of both known and hidden covariates remains a continuing challenge in the unsupervised analysis of single-cell genomics data.

We previously described an unsupervised computational approach called Iterative Clustering and Guide-gene Selection (ICGS), designed to discover both discrete and transitional cell populations from diverse scRNA-Seq platforms (Olsson *et al.*, 2016). ICGS iteratively identifies core variable gene expression programs from a cell/gene-expression matrix through multiple rounds of hybrid clustering (HOPACH; van der Laan and Pollard, 2003), selection of maximally informative guide-genes (transcription factor biased) and expression correlation. While ICGS was found to identify cell populations that could not be detected from alternative workflows (Churko *et al.*, 2018; Olsson *et al.*, 2016), this approach has several limitations that hinder its use in large-scale studies of tens or hundreds of thousands of cells. Notably, HOPACH clustering is computationally expensive with increasing dataset size, is not effective at partitioning datasets with dozens of discrete cell populations and cannot effectively distinguish between technical artifacts (e.g. doublet versus real cell clusters).

Here, we present the next iteration of ICGS. ICGS2 incorporates additional downstream methods to improve subtype detection, scale to extremely large and complex datasets and automate parameter estimation. To analyze extremely large datasets, while retaining rare cell populations, ICGS2 applies an intelligent sampling-based strategy for large scRNA-Seq datasets to capture the most informative cells for downstream unsupervised analyses. To improve the identification of both broad and rare cell clusters, ICGS applies a secondary sparse-non-negative matrix factorization (NMF) analysis (Kim and Park, 2007), automatically estimates *k*, excludes clusters with no uniquely expressed genes (cluster fitness) and assigns all cells to identified cell populations using support vector machine (SVM) classification (Cortes and Vapnik, 1995). This workflow is implemented as an easy-to-use automated pipeline through integration with the software AltAnalyze (Emig *et al.*, 2010). This workflow can be run from the command-line or an intuitive graphical user interface and includes a large repertoire of user-friendly integrated downstream analysis tools (e.g. cell-type prediction, differential expression, pathway analysis). We demonstrate improved performance of ICGS2 when compared to diverse alternative algorithms applied to scRNA-Seq datasets of varying size and complexity (e.g. donor-bias). Importantly, this approach remains scalable to ultra-large datasets (memory efficient), without sacrificing sensitivity.

## 2 Materials and methods

### 2.1 Algorithm design

**Fig. 1.**
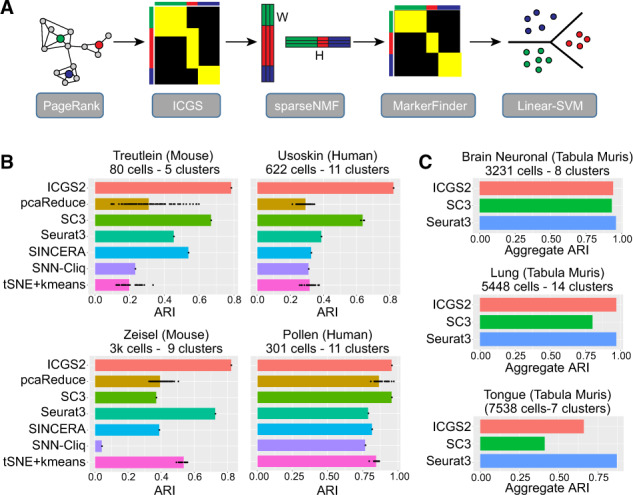
Performance of ICGS2 against diverse alternative unsupervised scRNA-Seq algorithms. (**A**) Overview of the ICGS2 workflow for single-cell RNA-Seq population prediction. These steps include: (i) PageRank-downsampling (optional), (ii) feature selection (ICGS), (iii) dimension reduction (SNMF), (iv) cluster refinement/exclusion (‘fitness’) and (v) cluster assignments (linear SVM). (**B**) Comparison of ICGS2 to previously evaluated algorithms and benchmarking datasets of varying size and complexity to detect prior-defined cell populations. Performance of each method was evaluated by comparing the author annotated cell-to-cluster assignments to those obtained by each algorithm using the ARI (Section 2). (**C**) Comparison of ICGS2 to the top performing methods for Tabula Muris tissue scRNA-Seq (SMARTSeq2) from panel B, using an aggregated ARI to account for contributing composite sub-clusters (see Supplementary Fig. S1B for corresponding ARI values and in Supplementary Table S1 for cluster numbers)

The software ICGS2 has been developed as part of an open-source python toolkit, AltAnalyze, with extensive documentation on its use, algorithms and optional user-defined parameters (https://altanalyze.readthedocs.io/en/latest/). ICGS2 identifies cell clusters through a five-step process: (i) PageRank-downsampling of cells (optional), (ii) feature selection (ICGS2), (iii) dimension reduction and clustering [sparse-NMF (SNMF)], (iv) cluster refinement (MarkerFinder algorithm) and (v) cluster reassignments (SVM) (Fig. 1A). AltAnalyze includes support for multiple input formats including: (i) an expression file, such as raw counts or counts-normalized, non-log or log2, with genes as rows and samples as columns, (ii) 10× Genomics (version 1.0–3.0) produced filtered sparse matrix results (.mtx, HDF5), (ii) genome-aligned BAM files or (iv) FASTQ files for individual cells. A tab-delimited gene-counts matrix can be normalized within the software or prior to import using the module CountsNormalize. The principle steps of this program are:

#### 2.1.1 Step 1: downsampling with PageRank (recommended for datasets with > 2500 cells or user-defined)

In this step, cells within a scRNA-Seq dataset are downsampled, to allow ICGS2 to run with a small memory footprint on datasets of varying sizes. For datasets of >2500 cells by default (user defined), PageRank alone is performed on a *k*-nearest neighbor graph of the cells, while for datasets of over 15 000 cells, initial downsampling is performed using a community clustering approach followed by PageRank:

- *Selection of variable genes for downsampling*: ICGS2 imports an input expression file processed from AltAnalyze (automatically normalized by cell total read counts and log2 transformation, for protein-coding genes and initial ICGS variance filtered) and identifies the top 500 genes with the highest dispersion (user defined). Excluded from this set are mitochondrial genome, L and S ribosomal genes and immunoglobins to minimize batch and donor effects (default option). Dispersion for each gene is calculated as the ratio of the variance divided by its mean. The resulting PageRank input file is filtered for these genes.
- *Graph construction*: A graph representation of the dataset (limited to the filtered genes from Step 1a) is created by using the cells as vertices and connecting cells with edges to the *k*-nearest neighbors (*k*=10 default) of each cell. The graph is created using the networkx python package and identification of the *k*-nearest neighbors is performed using the python package Annoy (Aumüller *et al.*, 2020).
- *PageRank*: Once the graph is generated, a score is calculated for each cell based on PageRank score (networkx python library). The Google PageRank algorithm (Page *et al.*, 1999) is a graph-based algorithm, originally designed to identify the most frequently visited websites. Since the graph is generated by connecting each cell to only its nearest 10 cells, cells from smaller populations can have high PageRank scores and thus be represented in the sampling. Thus, the approach prioritizes the selection of interspersed nodes in the larger graph, with minimum representation bias. PageRank has previously been evaluated for graph sampling and shown to perform comparatively better than other approaches (Leskovec and Faloutsos, 2006). In ICGS2, the top 2500 cells (by default) with the highest PageRank scores are selected and used for the remaining analysis (steps 2–5). For datasets of millions of cells, this default threshold would likely need to be increased by the user to accommodate potentially hundreds of cell types (downsample option).
- *Louvain-based downsampling*: For very large scRNA-Seq datasets (*n*>15 000 cells), an initial preliminary downsampling is performed using community detection via Louvain clustering (community python library), after graph construction and prior to PageRank.

Louvain-based downsampling is performed to reduce the cell space for PageRank (PageRank is not sufficiently scalable to ultra-large datasets). Louvain Clustering has become a standard approach to perform clustering single-cell datasets. Several tools, such as Monocle3 (Cao *et al.*, 2019), Seurat (Butler *et al.*, 2018) and Scanpy (Wolf *et al.*, 2018), use the approach as default. In ICGS2, Louvain clustering is performed with the lowest possible resolution (*r*=0) to find maximal clusters (smallest communities). This value indicates at which level to cut the clustering dendrogram, with 0 resulting in the most granular clusters. This approach helps sample an equal number of representative cells for rare and extremely large cell populations. For each community identified using Louvain clustering, *m* representative cells that have the smallest mean Euclidean distance to all other cells in that community (most central) are selected as representative cells of that community. The most representative cell for a community is defined as

(1) $$x_{\mathrm{representative}}={\mathrm{argmin}}_{y\in \{x_{1},x_{2},\ldots .x_{c}\}}\sum_{i=1}^{c}d\left(y,x_{i}\right),$$

where $x_{1},x_{2},\ldots .x_{c}$ are the cells of a community, *c* is the total number of cells in the community and *d* is the distance function (Euclidean). The number of cells to select as representatives for each community is defined from the maximum number of cells to initially downsample to (*s*), the total number of communities detected (*n*) and the number of cells in each community (*c_i_*). The total number of representative cells selected for community *i* is given by

*m_i_*=argmin(*c_i_*,$\frac{s}{n}$), for *i* = 1,2,3,…*n*, (2)

where *s*=10 000 cells by default (downsample_cutoff×4, where downsample_cutoff=2500 cells by default). In effect, this process leads to selecting (*s*/*n*) representatives for each community, except for those communities with fewer than the average community size. From these 10 000 downsampled cells (variable based on downsample_cutoff), PageRank is used to further downsample (2500 cells by default).

#### 2.1.2 Step 2: feature selection

While feature selection in ICGS2 is the same as in the original ICGS, the associated thresholds are now automatically determined, including the correlation cutoff appropriate for the dataset. In brief, ICGS identifies correlated gene modules through pairwise correlations of variable genes [Pearson correlation coefficient (rho) above a user supplied threshold (default = 0.2)], followed by multiple rounds of HOPACH clustering of genes and cells (the Guide3 file is generated in the final round of ICGS clustering) and determination of representative marker genes (guide-genes) for supervised correlation analysis. Guide-gene selection enables the exclusion of cell-cycle gene expression modules by exclusion of guide-associated cell-cycle genes prior to supervised correlation of those guide-genes. ICGS has shown to improve the delineation of rare transcriptionally distinct populations while minimizing ‘batch’ or donor-bias through the selection of highly coherent gene expression clusters derived through intra-correlation of genes (Lu *et al.*, 2018; Olsson *et al.*, 2016). ICGS2 begins with a default Pearson rho threshold of 0.2 for the identification of correlated genes; however, if the number of initial correlated genes is >5000, the rho cutoff is automatically incremented by 0.1 and the correlation step is reiterated until this cutoff is met. By default, only protein-coding genes are considered with exclusion of mitochondrial genome, L and S ribosomal genes. 10× Genomics data are automatically imported and normalized [counts per gene divided by the total counts per barcode multiplied by a 10 000−counts per ten thousand (CPTT)].

#### 2.1.3 Step 3: dimension reduction with SNMF

To improve the delineation of cell clusters following HOPACH clustering in ICGS, SNMF is applied to the clustered cell data to improve population detection. SNMF uses an L1-norm minimization and is solved using a fast non-negativity constrained least squares algorithm (Kim and Park, 2007). This approach is frequently used for clustering non-negative sparse datasets. To obtain consistent results across multiple runs, the initialization is performed using the standard approach, non-negative double singular value decomposition (Boutsidis and Gallopoulos, 2008). The Guide3 results from ICGS (‘ICGS’ output directory) are produced as previously described (HOPACH output from the last step of the guide-gene correlation analysis) (Olsson *et al.*, 2016). To estimate the rank of the matrix (i.e. clusters) for SNMF, the ICGS Guide3 matrix is *z*-score normalized and its eigenvalues are calculated. The number of clusters is estimated as 2×*k*, where *k* is determined by the number of eigenvalues that are significantly different with *P* < 0.001 from the Tracy–Widom distribution (Kiselev *et al.*, 2017) whose mean is $(\sqrt{g-1}$+$\sqrt{c}$)^2^ and standard deviation is:

(3) $$\left(\sqrt{g-1}+\sqrt{c}\right)\times {\left(\frac{1}{\sqrt{g-1}}+\frac{1}{\sqrt{c}}\right)}^{\frac{1}{3}},$$

where *g* is the number of genes and *c* is the number of cells (Kiselev *et al.*, 2017).

Dimension reduction is performed on the ICGS Guide3 results using SNMF, which is available in the ‘nimfa’ python package. Given an input matrix *c*×*g* where *c* is the number of cells and *g* is the number of genes, the SNMF factorization returns two matrices: the basis matrix, *W* with the dimensions *c*×*r*, where *c* is the number of cells and *r* is the number of ranks and the coefficient *H* matrix with the dimensions *g*×*r*, where *g* is the number of genes and *r* is the number of ranks. For each cell, its provisional assignment is based on its largest contribution in *W*. All the parameters are set to default as per the package except the rank.

#### 2.1.4 Step 4: marker gene selection (cluster fitness)

In some cases, the clusters identified in Step 3 will be weakly defined by unique gene expression. To identify rigorously defined cell clusters with unique gene expression for downstream cell-cluster assignment (all cells, not just downsampled), ICGS2 applies the MarkerFinder algorithm, which is a component of AltAnalyze (Olsson *et al.*, 2016). MarkerFinder identifies genes that are positively correlated with an idealized cluster-specific expression profile (1 or 0). For each SNMF cluster, a reference is created where cells belonging to the group are assigned 1 and the remaining cells are assigned 0. Each gene is correlated to the references and assigned to a particular cluster based on the highest Pearson correlation (rho). Using the initial correlation cutoff identified for ICGS pipeline, SNMF cell clusters with fewer than two genes above the supplied rho threshold are excluded from downstream analyses. As such, centroids will be derived for only clusters with unique gene expression for supervised assignment to those final clusters. The Top 60 Pearson correlated genes for each SNMF cluster with a rho >0.3 are considered for the remaining SNMF groups. As such, this method addresses the vital unmet need to exclude clusters that specifically result from doublet cell clusters with no uniquely expressed genes.

#### 2.1.5 Step 5: cell-cluster assignment (linear SVC)

(i) Using the marker genes identified for sufficiently fit clusters, cluster centroids are determined based on the cells assigned to the specific SNMF clusters. Next, a linear SVM model with a linear kernel is constructed. (ii) The SVM prediction model is applied to all the cells in the dataset and reclassified based on the training models. ICGS2 uses the linear SVC option in scikit-learn (default parameters). When evaluated, SVM has shown to perform well for single-cell datasets (Abdelaal *et al.*, 2019).

### 2.2 User parameters

By default, ICGS2 includes built-in automated parameter estimation for its correlation cutoff (ICGS and MarkerFinder), estimation of number of clusters (rank estimation for SNMF) and number of cells to downsample for PageRank. These defaults can be explicitly set by the user to force the software to identify more or fewer clusters/heterogeneity. Additionally, ICGS has default options which can be modified by the user including: (i) intra-gene variability ‘fold’-threshold (Step 2, ICGS), (ii) protein-coding gene filter (Step 2, default=yes), (iii) exclusion of cell-cycle effects, (iv) HOPACH clustering metric for columns (Step 2, default=Cosine; other options are Euclidean and correlation), (v) number of cells to downsample to (Step 1, default=2500) and (vi) exclude outlier cells (default=no; other options are yes and the minimum number of genes expressed with a CPTT >1 (default≥500). For evaluation of these methods, the software defaults have been used.

### 2.3 Cell-type prediction

ICGS2 automatically performs a gene-set enrichment analysis on each cell population marker gene cluster using the software GO-Elite (Zambon *et al.*, 2012) (see Fig. 4A). This database includes marker genes for tissues and purified cell types (Olsson *et al.*, 2016) and those previously curated from diverse published scRNA-Seq studies [e.g. Mouse Cell Atlas, Human Cell Atlas (HCA), fetal development]. Cell-type predictions are displayed on the resulting UMAP plot.

### 2.4 Software outputs

ICGS2 results include marker gene heatmaps with likely predicted cell types (downsampled and all cells), UMAP projection, unique marker genes associated with each cell population and ranks (text file), SVM scores (text file) and cell-to-cluster (text file) associations within the ICGS-NMF and NMF-SVM folders. Secondary results include predicted cell-population labels (GO-Elite), differential expression results between clusters, protein–protein and protein–DNA predicted interactions among these genes (network plots), QC plots, cellHarmony cell-type predictions (DePasquale *et al.*, 2019) and GO-Elite pathway/ontology/gene-set enrichments by default (Zambon *et al.*, 2012).

### 2.5 Benchmarking

To evaluate the performance of ICGS2, nine datasets were considered (Supplementary Methods). We compared ICGS2 clustering results to the cell-population labels determined by the authors of the different datasets tested. We use the Adjusted Rand Index (ARI) (Hubert and Arabie, 1985) which has been used previously benchmark other unsupervised scRNA-Seq subtype prediction algorithms. To maximize the ARI score for each approach, we calculated an aggregate ARI where if multiple clusters were predicted for a single reference population (high specificity >0.75), these clusters were combined prior to scoring, using a custom python script. The specificity for a tested cluster is given as

(4) $$\mathrm{Specificity}\;(si\;\mathrm{for}\;a\;\mathrm{given}\;j)=\frac{\mathrm{Number}\;\mathrm{of}\;\mathrm{cells}\;\mathrm{overlap}\;\mathrm{in}\;i\;\mathrm{and}\;j}{\mathrm{Number}\;\mathrm{of}\;\mathrm{cells}\;\mathrm{in}\;i}\times \;100,$$

where *i* represents the tested algorithm’s cluster and *j* is a ground truth cluster tested against. A detailed description of all benchmark datasets, parameters for algorithms tested (ICG2, Seurat3, SC3, Monocle3, CellSIUS) and the simple random sampling (SRS) procedure is provided in Supplementary Methods. Associated ICGS2 clustering results, input data files can be obtained at: https://www.synapse.org/#!Synapse:syn18659335.

## 3 Results

To improve the prediction of discrete cell populations from diverse possible single-cell RNA-Seq datasets, we developed a significantly improved iteration of our previously described software ICGS (Olsson *et al.*, 2016). These new methods were built on-top of ICGS rather than creating a new method from scratch, as this software has several potential fundamental advantages over existing approaches. These advantages include ease-of-use (graphical and non-graphical user interfaces), a lack of reliance on dimension reduction to identify initial cellular and gene expression heterogeneity (guide-gene-based discovery), automated data visualization outputs (heatmap, UMAP), methods for cell-type prediction and embedded pathway/network analyses. To improve the delineation of rare transcriptional cell populations, we have augmented the core ICGS algorithms with rigorous methods for determining biologically valid clusters (SNMF, SVC, cluster fitness), automated cluster number determination, introduced a new method for accurate downsampling (e.g. PageRank) for large scRNA-Seq datasets, added new methods for data visualization (UMAP) and significantly updated the original cell-type marker gene database (Fig. 1A and Section 2.5). These methods were designed to increase the sensitivity of ICGS to identify important rare cell populations in datasets with potentially hundreds of thousands of cells.

### 3.1 ICGS2 has improved performance over alternative algorithms for established benchmarks

To assess the performance of the full ICGS2 workflow in comparison to its individual components (ICGS version 1 and ICGS with NMF alone), we evaluated each against multiple silver-standard reference datasets. The datasets Zeisel *et al.* (2015), Pollen *et al.* (2014), Usoskin *et al.* (2015) and Treutlein *et al.* (2014) were selected particularly for their diversity of size and number of clusters. The ARI method was used to evaluate cluster similarity against the author provided labels, considered here as ground state truth. As a first test, we note that for all four datasets, ICGS2 had improved ARI scores over each of its intermediate outputs (Supplementary Fig. S1A).

To compare ICGS2 to alternative unsupervised approaches, we considered previously obtained ARI scores on these same evaluated datasets from the software SINCERA (Guo *et al.*, 2015), SNN-Cliq (Xu and Su, 2015) and t-SNE+K-means and pcaReduce (Kiselev *et al.*, 2017). New versions of SC3 (version 1.8) (Kiselev *et al.*, 2017) and Seurat (version 3) (Butler *et al.*, 2018) were further substituted for prior benchmarked versions of these tools. Comparison of these ARI measurements finds that ICGS2 collectively outperforms all other approaches tested for these small and medium sized scRNA-Seq datasets (Fig. 1B).

### 3.2 Optimized population discovery from large scRNA-Seq datasets

ICGS2 is dependent on HOPACH clustering and SNMF which are computationally expensive with increasing dataset size. As such, it is not immediately applicable to ultra-large datasets (*n* > 50 000 cells). Hence, we implemented a new method for intelligent downsampling of scRNA-Seq data, prior to SVM classification of the entirety of cells in a dataset. While approaches such as SC3 apply random downsampling, this procedure is likely to miss rare cell populations or require a large sampling fraction. Alternatively, a recent downsampling single-cell method (BigScale) applies a *k*-nearest neighbor approach that is effective at preserving heterogeneity in large scRNA-Seq datasets, but requires specifying the number of nearest neighbors a priori (Iacono *et al.*, 2018). To address this challenge, ICGS2 applies the Google PageRank algorithm to identify the top 2500 representatives cell profiles (by default) for large scRNA-Seq datasets. We evaluated the performance of the PageRank-based downsampling approach with ICGS2 using three medium sized datasets with prior curated clusters from the Tabula Muris project (Tabula Muris Consortium, 2018): tongue (*n* = 5448 cells), lung (*n* = 7538 cells) and brain (*n* = 3231 cells). ICGS2 produced results in these comparisons that were comparable to Seurat3, but improved over SC3 (Supplementary Fig. S1B). As the observed ARI scores were relatively low for all three datasets, we further maximized the ARI score using an aggregate ARI which produced generally similar rankings with improved overall performance (Fig. 1C and Section 2).

### 3.3 Identification of distinct hematopoietic subtypes in the HCA

**Fig. 2.**
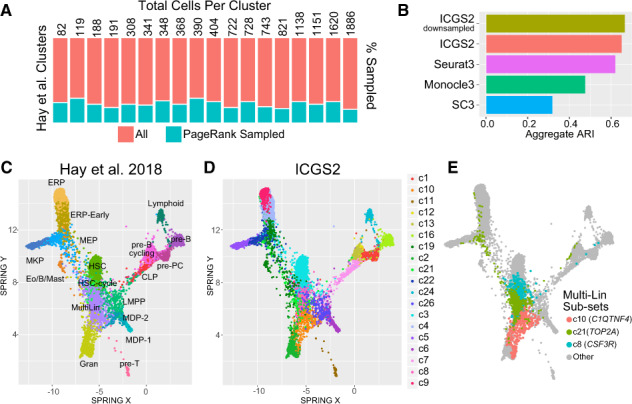
Delineation of discrete and transitioning populations in Bone Marrow Progenitors (BMP). (**A**) Frequency of PageRank-downsampled cells out of the total number of prior-defined HCA (*n* = 11 568) BMP clusters (*n* = 18). The sampled and total number of cells in each cell population are shown to the right of the plot. (**B**) Detection of prior annotated (Hay *et al.*, 2018) BMP clusters using ICGS2 and ICGS2 downsampled compared to Seurat3.0 with multiple-donor sample integration, SC3 and Monocle3, using aggregate cluster ARI. (**C**–**E**) Visualization of cell-cluster assignments using the software SPRING of: (C) prior-defined HCA BMP clusters, (D) ICGS2 using downsampling and (E) of novel multi-lineage populations identified uniquely by ICGS2. The top marker gene is shown for the three ICGS2 Multi-Lin clusters in parentheses. The number of original and aggregated clusters are provided in Supplementary Table S1

We recently performed a comprehensive analysis of eight independent donor bone marrow scRNA-Seq samples collected and profiled from HCA initiative (Hay *et al.*, 2018). This analysis defined 35 distinct hematopoietic cell populations from over 100 000 cells. Although the workflow applied in this analysis relied on ICGS version 1, ICGS was run independently on the cells from each eight donors individually, prior to those results being aggregated and used as references for cell alignment using the software cellHarmony (DePasquale *et al.*, 2019). This analysis produced both a combined dataset with all mature and progenitor cells and a separate analysis in which selectively defined and refined populations in presumptive bone marrow progenitors (BMPs) (11 548 CD34+ cells). We consider these predictions as additional ‘silver’ standards, as these populations were independently verified using prior sorted-population transcriptomic references, prior-defined cell-type marker genes and largely exclude donor-driven effects (Hay *et al.*, 2018). When comparing PageRank-downsampling of the selected 2500 cells from this dataset, the percentage of cells retained for each known group was consistently ∼17–26% of cells (22% total cells downsampled in the dataset) (Fig. 2A). Further, the results of the original ICGS2 and downsampled ICGS2 were highly concordant, with an aggregate ARI of 86% (ICGS2 downsampled compared to ICGS2 for all cells). When compared with SC3, Monocle3 (Cao *et al.*, 2019) and Seurat3 with multiple-donor sample integration, ICGS2 still had a higher or equivalent aggregated and non-aggregated ARI than these alternative methods (Fig. 2B and Supplementary Fig. S2A).

While none of the evaluated scRNA-Seq algorithms were able to identify several transcriptionally distinct clusters [two separate Monocytic Dendritic Precursor populations, Hematopoietic Stem Cell (HSC) in cycle versus HSC], both downsampled and all-cell ICGS2 analyses selectively identified common lymphoid progenitors and lymphoid-primed multipotent progenitors not identified by the other algorithms. ICGS2 further found additional granularity in the original annotated presumptive multi-lineage progenitor (Multi-Lin) cells (Fig. 2C–E and Supplementary Fig. S2B and C). While these Multi-Lin sub-clusters were also not identified using a specialized approach for rare sub-clusters identification (CellSIUS, see Supplementary Methods), this delineation is supported by unique gene expression present in these subsets with high expression of *CSF3R* and *SMIM24* (c8), *C1QTNF4* and *CSF1R* (c10) or cell-cycle genes (*TOP2A* and *MKI67*, c21) (Fig. 2E).

### 3.4 ICGS2 uniquely identifies novel sub-populations in ultra-large datasets with minimal donor effects

**Fig. 3.**
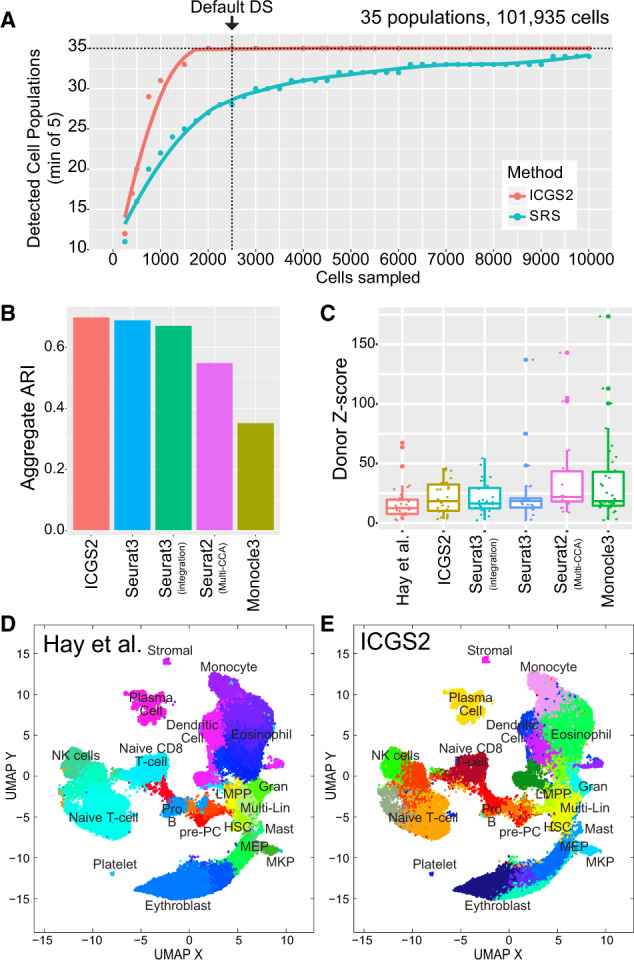
Identification of rare and novel cell populations from ultra-large scRNA-Seq data. (**A**) Comparison of ICGS2 downsampling and SRS at various thresholds to detect at least five cells in each of the 35 cell populations of the bone marrow HCA dataset. (**B**) Comparison of prior-defined bone marrow clusters (aggregate cluster ARI scores) using ICGS2 downsampled, Seurat3 (with and without the multi-donor integration workflow), Seurat2-Multi-CCA and Monocle. (**C**) Comparison of the different algorithms in detecting donor-biased bone marrow clusters (aka batch effects). Enrichment *z*-scores (Fisher’s Exact test) are calculated for each of the eight bone marrow donors against each cell cluster identified by the evaluated algorithm. A high *z*-score indicates an enrichment in cells in a specific cluster and a specific donor. (**D** and **E**) UMAP visualization of clusters for prior-defined bone marrow HCA scRNA-Seq clusters by Hay *et al.* (**D**) and by ICGS2 with downsampling (**E**). UMAP derived using Hay *et al.* marker genes. The number of original and aggregated clusters are provided in Supplementary Table S1

We next compared the performance of ICGS2 in the complete HCA bone marrow dataset (*n* = 101 618) against other approaches compatible with ultra-large scRNA-Seq datasets. For datasets of >15 000, Louvain clustering is performed on the *k*-nearest neighbor graph with the minimum resolution to more efficiently downsample the data to around 10 000 cells, prior to performing PageRank to identify the final top 2500 representative cells by default. Following downsampling, at least six representative cells per population were selected by this downsampling method for all 35 previously defined bone marrow cell populations (Supplementary Fig. S3A). ICGS2 was able to effectively sample cells from all 35 cell populations with 2500 selected cells as compared to SRS, which required ≥15 000 sampled cells (Fig. 3A and Supplementary Methods). To compare its ability to detect cell populations, ICGS2 was again evaluated relative to Seurat, Monocle3 and SC3, which have previously shown to effectively handle large scRNA-Seq datasets. To assess the contribution of donor-driven effects in the clusters obtained, Seurat3 was run with all samples combined (no batch effects correction) or by considering different donors using Seurat integration or canonical correlation analyses (Multi-CCA). While runtime on this dataset ranged from 80 min (Monocle3) to 7.5 h (Seurat3), ICGS2 proved to be the most memory efficient method, while remaining relatively fast (2 h) (Table 1). We attempted to run SC3, however, this approach reached its memory limit with 256 GB of RAM (estimate *k*-step) (see Section 2). BigScale was excluded from evaluation as it is currently compatible only with Windows operating systems with a Matlab license required. Even with downsampling, the aggregated ARI of ICGS2 was comparable to that of Seurat3 (with and without integration) and improved over Seurat-Multi-CCA and Monocle3 (Fig. 3B and Supplementary Fig. S3B). This included the detection of exceedingly rare populations by ICGS2 (e.g. CD34+ eosinophil, stromal and platelet). In addition, ICGS2 and Seurat3 with integration identified clusters that were least confounded by donor effects, including those identified by Seurat-Multi-CCA (Fig. 3C). In addition to the previously described bone marrow cell populations, ICGS2 uniquely identified distinctive additional subtypes of T cells, Erythroblasts and Dendritic cells (DC) which were not previously identified (Fig. 3D and E and Supplementary Fig. S3C–G). For example, each DC cell cluster was found to expresses unique marker genes with established roles in functionally distinct DC subsets (plasmacytoid, maturing CD1c+, CD1c+ and CD8+) (Supplementary Fig. S3H–K) (Cao *et al.*, 2006; Eggink *et al.*, 2018; Heger *et al.*, 2018; Orabona *et al.*, 2006; Yan *et al.*, 2016). It is important to note that most approaches failed to sufficiently define all of the discrete CD34+ cell populations in the entire HCA dataset that were clearly resolved from the independent analysis of these cells, with Seurat3 (integration) also finding many of the same novel ICGS2 populations (Supplementary Fig. S3C–G). Nonetheless, ICGS2 outperformed or was equivalent to these other approaches and identifies unique cell populations that align to prior knowledge.

**Table 1.** Benchmarking of ICGS2 and alternative approaches

Application	Maximum memory (GB)	Processing time (min)
ICGS2	10	121
Monocle3	170	81
Seruat3	116	441
Seruat3 integration	79	455

**Fig. 4.**
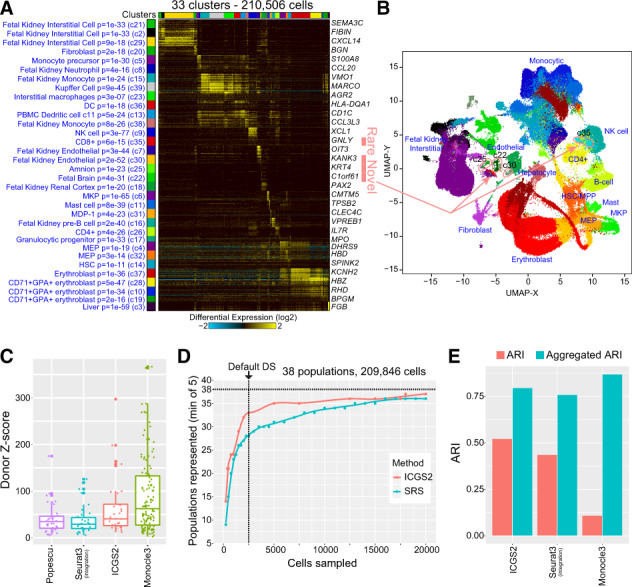
Identification novel cell populations in fetal hematopoiesis. (**A**) ICGS2 produced heatmap of identified cell populations among ∼210 000 cells from 15 different fetuses from liver, kidney and skin. The blue text indicates enriched cell-type markers from the default gene-set enrichment analysis with the top default displayed markers for each cell population shown the right of the heatmap. (**B**) UMAP visualization of cell clusters corresponding to panel A. (**C**) Comparison of the different algorithms in detecting donor-biased clusters (aka batch effects). Enrichment *z*-scores (Fisher’s Exact test) are calculated for each of the 24 fetus/tissues against each cell cluster identified by the evaluated algorithm (see Fig. 3C). (**D**) Comparison of ICGS2 downsampling and SR at various thresholds to detect at least five cells in each of the author annotated fetal hematopoiesis cell populations (*n* = 38, with doublet clusters). (**E**) Comparison of prior-defined fetal hematopoiesis clusters (ARI scores and aggregated ARI) using ICGS2 downsampled, Seurat3 (with multi-donor integration workflow) and Monocle3. The number of original and aggregated clusters are provided in Supplementary Table S1

As a final evaluation of ICGS2, we reanalyzed a large human scRNA-Seq dataset of fetal hematopoiesis from 15 different embryo/fetuses ranging in age from 7 to 17 weeks of gestation (Popescu *et al.*, 2019). This dataset has ∼210 000 cells from liver, kidney and skin, using CD45+, CD45- or no selection, with 38 cell clusters originally derived from separate tissue-donor integrative analyses (four predicted doublet clusters). ICGS2 analysis of all cells identified 33 cell populations, with relatively minimal suggested donor effects in comparison to the author provided labels (Fig. 4A–C). Comparison of ICGS2 downsampling with Louvain and PageRank selection, sampled five more author annotated cell populations than SRS at the default 2500 cell cutoff (Fig. 4D). Although only 2500 downsampled cells were used by ICGS2, cell-type annotations from ICGS2 biomarker gene-set enrichment aligned largely to the original authors 38 cell populations, but with very important distinctions (Fig. 4A and B). First, ICGS2 did not identify any of the prior annotated doublet clusters. Second, ICGS2 uniquely identifies ultra-rare populations not described by the original authors, including neuronal and endothelial cell populations with only <800 cells (<0.05%), largely corresponding to the authors’ ‘non-immune’ population. Importantly, we were able to find independent evidence for three out of four novel rare cell populations with Monocle3 (neuronal and endothelial), suggesting these are indeed real. While Monocle3 had the greatest aggregated ARI when using the default of 100 dimensions (recommended for large scRNA-Seq datasets), it also possessed the lowest non-aggregated ARI, due to the large number of output cell populations (*n* = 135), as Monocle3 requires the user to estimate the target number of clusters (Fig. 4E).

## 4 Discussion

As scRNA-Seq approaches continue to increase in the depth of cells captured and molecules measured, more sensitive approaches are required to identify rare and subtly distinct cell populations associated unique gene expression programs. Here, we present an improved and highly scalable version of ICGS, that can be applied to extremely large scRNA-Seq datasets to delineate subtly distinct and rare cell populations. We use a hybrid approach that combines accurate methods for cluster determination and cell classification, in combination with new approach for intelligent single-cell downsampling. NMF has been shown to improve the detection of sub-populations from diverse datasets, due to its ability to identify interpretable parts from high dimensional datasets (Mejía-Roa *et al.*, 2015). Using this refined workflow, we demonstrate improved performance over a large spectrum of existing approaches, across different datasets of varying complexity and size. Importantly, the use of iterative gene correlation and guide-gene selection appears to significantly minimize the impact of donor effects in ultra-large scRNA-Seq datasets, without directly considering such effects. This approach further uniquely identifies novel cell populations in bone marrow and fetal hematopoiesis that decompose multiple prior-defined cell-types associated with biologically informative markers (Multi-Lin, T cells, Erythroblasts and DC).

ICGS2 is fundamentally distinct from alternative approaches in terms of its basic strategy to identify heterogeneity. Standard methods for variable gene selection (dispersion, PCA) are inherently more susceptible to initial transcriptional noise, batch and donor effects; however, ICGS selects variable genes through a rigorous pairwise correlation strategy over multiple rounds of iteration, with a focus on the selection of transcription factors as guide-genes. As previously demonstrated, this approach is more likely to identify transitional states which include mixed-lineage progenitors, weekly defined by unique gene expression (Hay *et al.*, 2018; Hulin *et al.*, 2019; Lu *et al.*, 2018; Magella *et al.*, 2018; Olsson *et al.*, 2016; Yáñez *et al.*, 2017). ICGS2 extends the ability of ICGS to further define rare and common transcriptionally distinct populations, including multi-lineage cell populations from the HCA, independent of donor effects. Because the software automatically identifies the most appropriate number of clusters, it can be simultaneously applied to many datasets, without the requirement for the user to specify.

The potential applications of this approach are broad, which include emerging large-scale whole-organism atlases, where AltAnalyze provides additional advantages beyond the ICGS2 algorithm itself. These benefits include imbedded methods to predict cell-type identify based on existing cell-specific gene-set references (gene-set enrichment, cellHarmony, pathway enrichment analysis) and display of protein–protein and transcriptional regulatory network relationships among genes differentially expressed between similar populations (NetPerspective algorithm) (Lu *et al.*, 2018). Importantly, this workflow is accessible by both knowledgeable single-cell data analysts as well as conventional biologists without such expertise, through accessible command-line and graphical user interfaces. An important caveat of this approach is that it is dependent on the presence of coordinated gene expression patterns in which the underlying data are not so sparse that initially correlated genes can be identified. To address this challenge, this tool further includes the ability for users to designate the number of clusters when initial heterogeneity is only weakly detected. While the parameters of ICGS2 and other methods (e.g. SC3, Seurat) can be modified to identify additional subtypes; in the future, we hope to optimize our approach to optionally find maximal heterogeneity at the lowest resolution (sub-clustering). Through similar uses of ICGS2, we anticipate the discovery novel biologically informative cell populations that can guide our understanding of cellular diversity on complex organisms, including exceedingly rare populations that underlie disease phenotypes.

## Supplementary Material

btaa201_Supplementary_Data
