# Supplementary material for: Resolving single-cell heterogeneity from hundreds of thousands of cells through sequential hybrid clustering and NMF
Source: Bioinformatics. 2020 Mar 24;36(12):3773–80. doi: 10.1093/bioinformatics/btaa201 (PMC7320606; doi:10.1093/bioinformatics/btaa201)
Supplement: btaa201_Supplementary_Data [file btaa201_supplementary_data.zip › btaa201-Suppl_Data/Supplemental Information.pdf]

Supplemental Information

Resolving single-cell heterogeneity from hundreds of thousands of cells through sequential hybrid clustering and NMF

Meenakshi Venkatasubramanian[1,2], Kashish Chetal[2], Daniel Schnell[2], Gowtham Atluri[1], Nathan Salomonis[2,3,4]

[1]Department of Electrical Engineering and Computer Science, University of Cincinnati, Cincinnati, OH

[2]Division of Biomedical Informatics, Cincinnati Children's Hospital Medical Center, Cincinnati, OH

[3]Department of Pediatrics, University of Cincinnati School of Medicine, Cincinnati, Ohio, USA.

[4]Department of Biomedical Informatics, University of Cincinnati, Cincinnati, OH

Contents

1 Extended Methods *3*

1.1 Dependencies 3

1.2 Command-line Options 3

1.3 Simple Random Sampling Procedure 4

1.4 Dataset Evaluation 4

2 Supplementary Figures *8*

2.1 Figure S1 8

2.2 Figure S2 9

2.3 Figure S3 10

3 References *12*

1 Extended Methods

1.1 Dependencies

ICGS2 requires the following dependencies:

- Python 2.7+: numpy, scipy, matplotlib, sklearn (scikit-learn), nimfa, numba, python-louvain, annoy, networkx, fastcluster
- R version 3+: biocLite, hopach

These dependencies will be installed automatically when installing AltAnlayze from PyPI:

- command: pip install altanalyze

1.2 Command-line Options

ICGS2 can be run directly from the AltAnalyze graphical user interface (version 2.1.3+) or from the command-line. From the command-line, users will typically specify an input tab-delimited expression files (scaled and log2 normalized) or an input 10x Genomics file. Two input file formats are supported for 10x Genomics; 1) matrix files (.mtx or .mtx.gz) or 2) HDF5 (.h5). The below example is using a 10x Genomics input file:

$H5 = the directory path of a h5 or .mtx format sparse matrix file
$OUT = Output directory path (must already exist)

python AltAnalyze.py --platform RNASeq --species Hs --ChromiumSparseMatrix $H5 --expname "MyDataset" --output $OUT --runICGS yes

Optional command-line flags to include:

--restrictBy None (includes ribosomal, mitochondrial and non-coding genes [default=protein_coding])
--excludeCellCycle conservative (excludes discovered cell-cycle correlated guide-genes [default=no])
--removeOutliers yes (excludes cells with < 500 genes expressed [numGenesExp] by default)
--numGenesExp 200 (see the above option [default=500])
--downsample 5000 (number of cells for louvain/PageRank downsampling [default=2500])
--FoldDiff 1.5 (value used for variance filtering. Higher values increase the stringency [default=4])
--SamplesDiffering 20 (cell cluster minimum size [default=4])
--row_method ward (cluster algorithm to apply for ICGS row clustering [default=hopach])
--column_metric euclidean (clustering algorithm to apply for ICGS column clustering [default=cosine])

--k 100 (increase or decrease the target number of NMF clusters [default=None]

When supplying a text file rather than sparse-matrix as input replace --ChromiumSparseMatrix with --expdir (path of the log2 read normalized counts file - tab-delimited text file format).

1.3 Simple Random Sampling Procedure

To evaluate the ability of ICGS2 to identify distinct rare and common populations from ultra-large scRNA-Seq dataset, ICGS2 was compared to simple random sampling (SRS) of the data for the same number of down-sampled cells. For both ICGS2 and SRS, we used n=5 as the minimum number of cells from a population needed in a sample in order to classify the population as represented in a sample. Random samples (without replacement) were drawn from the cell label vector, with sample sizes ranging from 200 or 250 cells to an upper limit that depended on the dataset. 1000 random samples were drawn for each sample size. For each random sample, the number of cells drawn from each population was tallied. Across the 1000 random samples, the 5th percentile of the count distribution was determined for each population. If the 5th percentile of the count distribution of met or exceeded the 5-cell minimum, that population was considered "represented" by the SRS method. Roughly speaking, a "represented" population means that 95% or more of the 1000 SRS replicates (for a given sample size) had 5 or more cells from the population. We also explored using the 10th percentile of the count distribution and obtained nearly identical results.

1.4 Dataset Evaluation

Evaluated Datasets

1. SC3 prior benchmarked datasets. ARI scores, cell-to-cluster assignments and expression data were obtained from a prior evaluation study (Kiselev, et al., 2017). The evaluated datasets used in the evaluations were from Zeisel (Zeisel, et al., 2015), Pollen (Pollen, et al., 2014), Usoskin (Usoskin, et al., 2015) and Treutlein (Treutlein, et al., 2014). This included evaluation of the algorithms SINCERA (Guo, et al., 2015), SNN-Cliq (Xu and Su, 2015), tSNE+kmeans and pcaReduce. A newer version of SC3 v.1.8 (Kiselev, et al., 2017) and SEURAT v.3.0 were evaluated in place of the prior earlier versions of these software.

2. Mouse Tabula Muris project single-cell analysis. Three scRNA-Seq tissue datasets were selected from the Tabula Muris consortium analysis of 20 different organ systems using SMARTSeq2 and 10x Genomics technologies (Tabula Muris, et al., 2018). Standardized cell-

type annotations were assigned by the authors using Cell Ontology definitions. These SMARTSeq2 datasets were selected on the basis of the following criterion: 1) cell counts > 3,000 and 2) 7 or more author annotated cell populations. These tissues consisted of brain (3,231 cells, 8 annotated cell populations), lung (5,448 cells, n=14 annotated cell populations) and tongue (7,538 cells, n=7 annotated cell populations). For Brain, only the non-immunological cell population dataset was evaluated. Only cells passing the ICGS default outlier removal filter (>500 genes expressed) were evaluated for all analyses.

3. Human Bone Marrow. 10x Genomics scRNA-Seq data from eight independent donor bone marrow biopsies collected and profiled from Human Cell Atlas (HCA) initiative (Group, 2018; Hay, et al., 2018). Both counts and AltAnalyze normalized CPTT values were used as input for the below described evaluation algorithms. Although multiple captures were performed for each sample, cell profiles were aggregated on a per donor basis prior to analysis (i.e., for Seurat3 integration analysis). Evaluations were performed with either previously defined presumptive bone marrow progenitors (11,548 CD34+ cell clusters [n=18]) and all union of all analyzed donor cells (~100,000 cells, 35 originally annotated cell clusters).

4. Fetal hematopoiesis. 10x Genomics scRNA-Seq data from human fetal liver, skin, kidney and yolk sac cells from 7-17 weeks of gestation was obtained from a prior publication along with provided manual cell annotations (Popescu, et al., 2019). This dataset consisted of 210,506 cells, including putative doublets, with a total of 39 annotated cell populations (after correction of a spelling errors). One putative doublet cell population with only 14 cells, Erythroid Mac, was excluded. Unique gene expression was found in all cell populations, with the exception of Erythroid-Mac, Mono-NK and MEP and were deemed to be likely doublets or miss-annotations (MarkerFinder Pearson rho<0.3). Only cells passing the ICGS default outlier removal filter (>500 genes expressed) were evaluated for all analyses (n=3,974 excluded).

<u>Evaluated Algorithms</u>

For the evaluation of each algorithm, defaults were used per the recommendation of each tool and are otherwise specified below. We note that each package (default settings) scales each entry

in the count matrix by a factor to adjust for library size, followed by a log(1+x) transformation. Further, we applied the default normalization and feature selection methods for each application, rather than apply a standardized set of normalization and feature selection options, to be faithful to the original approach.

1. ICGS2. ICGS2 was run with default parameters for all the evaluations performed. The down-sampling cutoff was set to 2500 cells for all the analyses except for the Zeisel (n=3013 cells) and the Bone Marrow Progenitor dataset (11,548 cells) analyses, where the down-sample cutoff was set to value greater than the number of cells in the dataset. To compare ICGS2 to each of separate components, ICGS2 was run without down-sampling to obtain an intermediate HOPACH clustering results file (ICGS version 1.0) output prior to downstream dimension reduction analyses. To evaluate the use of SNMF, following this HOPACH derived clustering, the direct output of the SNMF step, without eliminating clusters without identifiable marker genes (cluster fitness – Step 4 above) and without individual SVM cell classification. ICGS down-sampling was compared to simple random sampling as described above.

2. Seurat3. For datasets with no batch or donor effects, Seurat3 (version3.1.0) was run without integration. Counts data was provided as input for each dataset. No additional genes or cells were filtered beyond those initial filtered using AltAnalyze for consistent evaluation. In brief, the scRNA-Seq data is normalized using the “LogNomalize” option as scaled to 10,000 (default). The feature selection was performed to identify the top 2000 most variable genes using “vst” option. PCA based dimension reduction using the most variable genes (n=2000). The top 20 PCs were used to find neighbors and Louvain clustering is performed at a resolution = 0.5 (“FindClusters”). When Seurat was run with integration the following values for parameters were used: The first 30 dimensions were considered for PCA, UMAP and to find neighbors. Louvain clustering was performed with a resolution cutoff=0.5. For the Fetal Hematopoiesis dataset, the 69 files could not be directly integrated due to too few cells in some of the individual datasets (anchor error). Hence, for both the fetal and adult hematopoiesis datasets, individual technical replicate 10x Genomics files for a single fetus or donor were combined into a single sample prior to integration (24 unified samples in the fetus and 8 in the adult bone marrow).

3. Seurat CCA. Individual counts file associated with different donors were provided as input files. The default normalization, scaling and finding variable genes options were called. 18 dimensions were considered for the functions “CalcVarExpRatio”,” AlignSubspace”, “RunTSNE” and “FindClusters”. Louvain clustering was performed using a resolution =0.5.

4. Monocle 3. A single combined counts file all donor/fetal samples was used as input for Monocle (no distinction between cells from different donors). 20 dimensions were considered for dimension reduction using UMAP. Louvain clustering was performed on the datasets using a resolution=1e-4. The Fetal Hematopoiesis dataset was tested with a dimension reduction = 100 as recommended by the authors for extremely large datasets.

5. SC3. SC3 requires both the counts and log normalized data as inputs. The estimate k function was run to determine the optimal k. Clustering was performed for each dataset with the ks value set to the optimal k determined. For datasets with > 5,000 cells, random sampling was performed to identify 5,000 cells to be used for clustering. The remaining samples were assigned into clusters using SVM classification. Note, that SC3 was not able to be run on datasets of 100,000 cells or greater, as the software reached its memory limit with 256GB of RAM (error during the estimate k-step). Although SC3 could be run using a range of k values, such options did not fit with the indicated evaluation criterion needed to independently estimate k.

6. CellSIUS. The CellSIUS workflow is an unsupervised computational method applied to scRNA-Seq datasets in which initial broad clusters have already been identified. This software was run according to the author recommendations in the published vignette, using the program defaults (Wegmann, et al., 2019). Although CellSIUS was applied to three datasets: 1) HCA bone marrow progenitors, 2) the unfiltered HCA bone marrow compendium and 3) to a subset of the fetal hematopoiesis dataset (~50,000 author annotated non-hematopoietic cells), only the HCA bone marrow progenitors ran without memory errors on a compute node with 256GB of RAM, with a noted limit of detection for < 25,000 cells. CellSIUS identified several extremely small clusters within the original author annotated Gran (n=18), MKP (n=51) and Pre-PC (n=34), with only partial support for this novel Gran cluster by Seurat3 (59% overlap, not shown).

2 Supplementary Figures

2.1 Figure S1

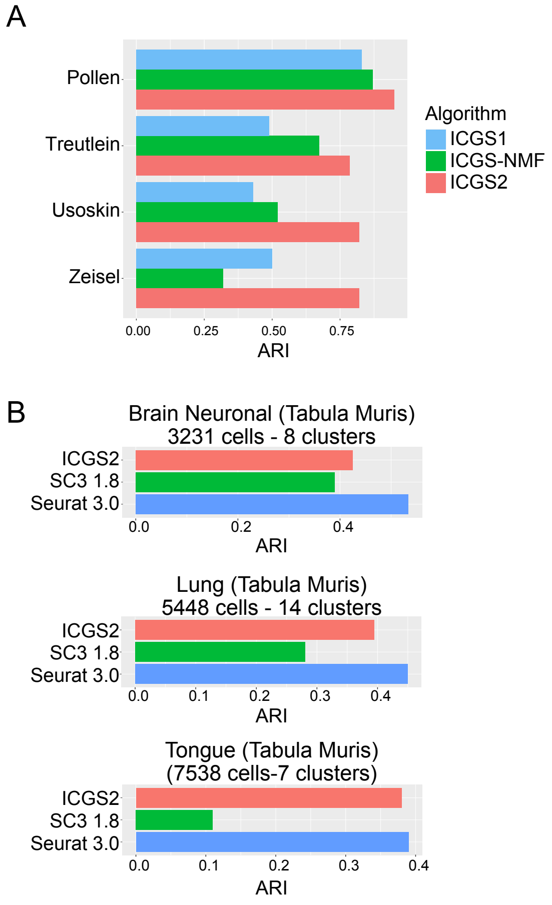

Figure S1. Evaluation of separate ICGS2 components. A) Comparison ICGS2 to ICGS version 1 results (without down-sampling) and to ICGS with NMF along (without cluster filtering or SVM) using the Adjusted Rand Index (ARI), for three prior benchmarked scRNA-Seq datasets. B) ARI values for ICGS2, SC3 and Seurat3 clusters prior to cluster aggregation. The number of original and aggregated clusters are provided in Table S1.

2.2 Figure S2

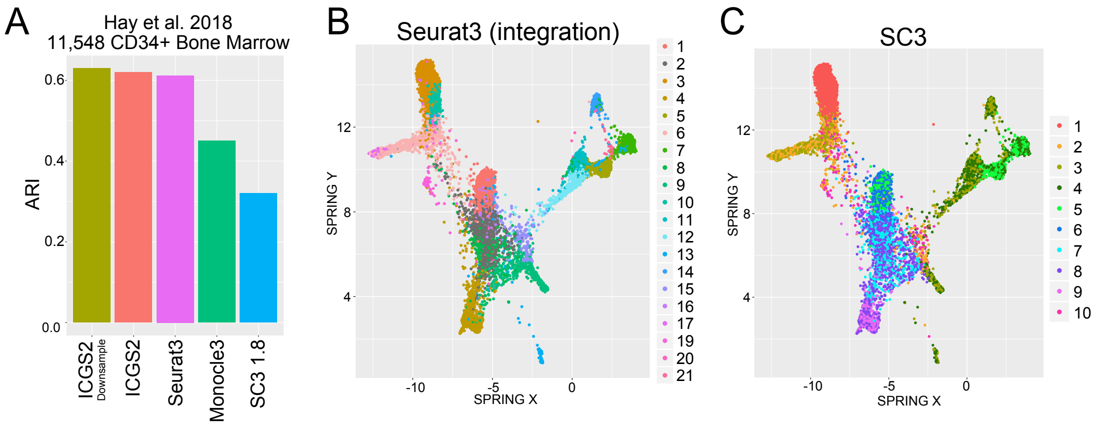

Figure S2. Evaluation of ICGS2 in human bone marrow progenitors.

A) Detection of prior annotated (Hay et al. 2018) bone marrow progenitor (BMPs) clusters using ICGS2 and ICGS2 down-sampled compared to Seurat 3.0 with multiple donor sample integration, SC3 and Monocle3 using non-aggregated ARI. B-C) Cell clusters obtained for BMPs from (B) Seurat3 with integration and (C) SC3, viewed on the original study author's SPRING plot (see Fig. 2C-E).

2.3 Figure S3

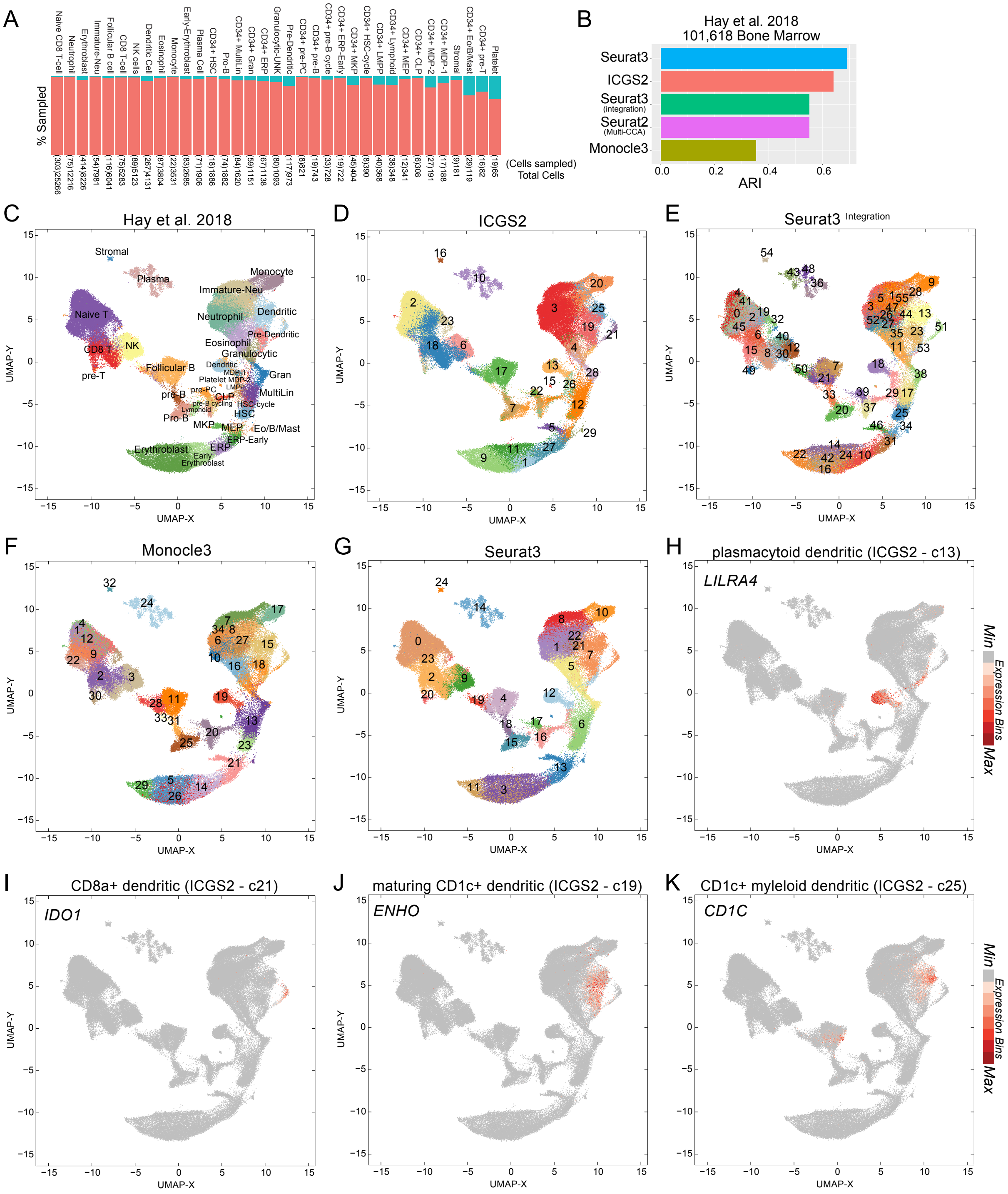

Figure S3. Comparison of rare and novel cell populations from ultra-large scRNA-Seq data.

A) The frequency of PageRank down-sampled Bone Marrow cells (n=100,514) from the Human Cell Atlas (HCA) project are shown relative to the total number of cells prior to sampling for 35 prior defined cell-

populations. The total number of cells in each cell population are shown to the right of the plot with the number of sampled samples in parentheses. B) Comparison of prior-defined Bone Marrow clusters (non-aggregated cluster ARI scores) using ICGS2 down-sampled, Seurat3 (with and without the multi-donor integration workflow), Seurat2 Multi-CCA and Monocle. C-K) UMAP visualization of 101,618 bone marrow cells for the top 50 ICGS2 marker genes, with population detection algorithm and/or displayed marker genes (produced in AltAnalyze). H-K) Four ICGS2 identified novel dendritic cell populations visualized using the top ranked ICGS2 gene marker.

3 References

Group, H.C.A.W. HCA Data Coordination Platform. In.; 2018.
Guo, M., *et al.* SINCERA: A Pipeline for Single-Cell RNA-Seq Profiling Analysis. *PLOS Computational Biology* 2015;11(11):e1004575.
Hay, S., *et al.* The Human Cell Atlas bone marrow single-cell interactive web portal. *Exp. Hematol.* 2018;68:51-61
Kiselev, V.Y., *et al.* SC3: consensus clustering of single-cell RNA-seq data. *Nature Methods* 2017;14(5):483-486.
Pollen, A.A., *et al.* Low-coverage single-cell mRNA sequencing reveals cellular heterogeneity and activated signaling pathways in developing cerebral cortex. *Nature Biotechnology* 2014;32(10):1053-1058.
Popescu, D.M., *et al.* Decoding human fetal liver haematopoiesis. *Nature* 2019;574(7778):365-371.
Tabula Muris, C., *et al.* Single-cell transcriptomics of 20 mouse organs creates a Tabula Muris. *Nature* 2018;562(7727):367-372.
Treutlein, B., *et al.* Reconstructing lineage hierarchies of the distal lung epithelium using single-cell RNA-seq. *Nature* 2014;509(7500):371-375.
Usoskin, D., *et al.* Unbiased classification of sensory neuron types by large-scale single-cell RNA sequencing. *Nat. Neurosci.* 2015;18(1):145-153.
Wegmann, R., *et al.* CellSIUS provides sensitive and specific detection of rare cell populations from complex single-cell RNA-seq data. *Genome Biol* 2019;20(1):142.
Xu, C. and Su, Z. Identification of cell types from single-cell transcriptomes using a novel clustering method. *Bioinformatics* 2015;31(12):1974-1980.
Zeisel, A., *et al.* Brain structure. Cell types in the mouse cortex and hippocampus revealed by single-cell RNA-seq. *Science* 2015;347(6226):1138-1142.
